# Angina Bullosa Hemorrhagica of the Ventral Tongue: A Case Report and Literature Review

**DOI:** 10.1155/crid/4477755

**Published:** 2026-08-31

**Authors:** Sara Akeel

**Affiliations:** ^1^ Department of Oral Diagnostic Sciences, Faculty of Dentistry, King Abdulaziz University, Jeddah, Saudi Arabia, kau.edu.sa

**Keywords:** angina bullosa hemorrhagica, case report, diagnosis, differential, hemorrhage, tongue diseases

## Abstract

Angina bullosa hemorrhagica (ABH) is an abruptly developing oral blood blister that most often affects the soft palate. Ventral‐tongue presentations are uncommon and can mimic traumatic, vascular, hematologic, or immune‐mediated disease. A 78‐year‐old Saudi man with hypertension and Type 2 diabetes presented with a 3‐day history of tongue discomfort and a solitary hemorrhagic bulla measuring 1.5 × 1.0 cm on the right ventral tongue. No other oral lesions, cutaneous involvement, or airway symptoms were present, and he reported a previous similar palatal lesion. Based on the characteristic clinical appearance, oral‐only distribution, and prior recurrence, a clinical diagnosis of ABH was made. The lesion was managed conservatively. Four days later, it had ruptured. At 2‐month follow‐up, the lesion had completely healed without scarring. This case highlights an uncommon ventral‐tongue presentation of ABH and emphasizes that ABH should be considered in the differential diagnosis of solitary hemorrhagic bullae and postbullous erosions of the tongue. Recognition of its characteristic clinical course may prevent misdiagnosis and unnecessary intervention while ensuring appropriate hematologic evaluation and, when indicated, investigation for immune‐mediated disease.

## 1. Introduction

Angina bullosa hemorrhagica (ABH) is a benign oral disorder characterized by the sudden development of blood‐filled blisters in the oral cavity or oropharynx without an identifiable hematologic, immunobullous, or systemic cause [1–5]. The lesions are typically solitary, dark red to purple, and short‐lived. They commonly rupture spontaneously, leaving a superficial erosion or ulcer that heals without scarring [1, 3–5].

The pathogenesis of ABH is not fully understood, although minor local trauma is considered an important precipitating factor. Reported triggers include mastication, hard or hot foods, dental procedures, airway instrumentation, endoscopy, and prolonged use of inhaled corticosteroids [1, 4–13]. Associations with diabetes mellitus and hypertension have also been described, although causality remains uncertain [5, 8, 14–16]. Although most cases resolve spontaneously without sequelae, large or posterior lesions may rarely cause airway compromise [4, 6–8, 11].

The soft palate is the classic site of involvement, but lesions may also occur on the buccal mucosa, tongue, floor‐of‐mouth, lips, uvula, pharynx, and other oropharyngeal sites [1, 3–5, 8, 11, 16–18]. Because tongue lesions are less typical than palatal lesions, they may be confused with traumatic hematoma, vascular lesions, pemphigus vulgaris, mucous membrane pemphigoid, bullous lichen planus, or hematologic disease [4, 5, 10, 13, 14, 17]. The present case is notable because it documents the sequential clinical evolution of an intact ventral‐tongue hemorrhagic bulla, its spontaneous rupture, and complete healing without scarring. We report this case to highlight an uncommon anatomical presentation of ABH and to review tongue and floor‐of‐mouth cases relevant to clinical diagnosis and differential assessment.

## 2. Case Presentation

A 78‐year‐old Saudi man presented to an oral medicine clinic with a 3‐day history of tongue discomfort. He was not concerned about the lesion and mainly wished to proceed with his planned dental treatment. He reported no pain and could not recall any preceding injury or other precipitating event. His medical history included hypertension and Type 2 diabetes mellitus, for which he was taking perindopril 5 mg, metformin 500 mg, linagliptin 5 mg, and gliclazide 30 mg. His dental history included restorative treatment and previous dental extraction. He was not taking antiplatelet or anticoagulant medication. He was a nonsmoker, did not consume alcohol, and had no relevant family history.

**Figure 1:**
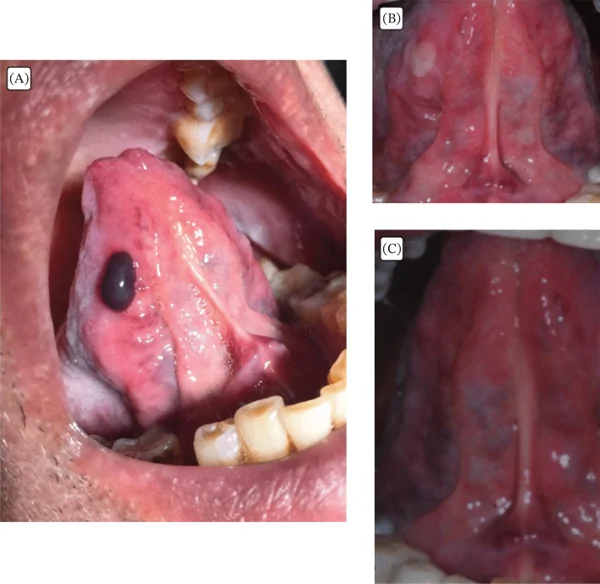
Sequential clinical evolution of angina bullosa hemorrhagica on the right ventral tongue. (A) Intact solitary hemorrhagic bulla at initial presentation measuring approximately 1.5 × 1.0 cm. (B) Postrupture ulceration approximately 4 days later. (C) Complete healing without clinically visible scarring at 2‐month follow‐up.

Extraoral examination was unremarkable. Intraoral examination revealed a fissured tongue and a solitary, well‐defined, sessile, fluctuant red‐to‐dark hemorrhagic bulla on the right ventral tongue measuring approximately 1.5 × 1.0 cm (Figure 1A). The lesion was nonpulsatile and no other oral lesions were identified. The patient reported a previous similar lesion on the palate, approximately 10 days earlier, although he could not recall the exact date and no photograph was available. Examination of the palate showed no residual ulceration or scarring. There were no cutaneous lesions, unexplained bruising or hematomas, or other evidence of extraoral mucocutaneous involvement.

The abrupt onset, characteristic hemorrhagic appearance, exclusively oral distribution, history of a similar palatal lesion, and absence of clinical features suggesting a generalized mucocutaneous disorder supported a clinical diagnosis of ABH. Because the lesion was localized, the patient was clinically stable, and there were no symptoms of airway compromise, conservative management was selected. The patient was reassured and advised to avoid local trauma and irritating foods. Four days later, he returned reporting spontaneous rupture of the lesion. Clinical examination showed a 1 × 1 cm ulcerated area at the same site (Figure 1B). He reported mild pain after rupture that diminished with time. At 2‐month follow‐up, there was complete healing without clinically visible scarring (Figure 1C). At the same visit, the patient reported another hemorrhagic bulla on the palate approximately 1 month after the index tongue lesion. The patient reported no cutaneous blisters, unexplained hematomas, or other similar lesions during follow‐up. Although the active lesion had not been examined, clinical examination at follow‐up showed no residual palatal ulceration or scarring (Table 1). A previous complete blood count, including the platelet count, was within normal limits; coagulation studies (PT/INR and aPTT) were not available. However, the patient was under regular medical and dental follow‐up and had undergone previous dental extraction without bleeding complications. Biopsy and direct immunofluorescence were not performed. A clinical diagnosis of ABH was made because six of the nine Ordioni criteria, including the two principal criteria, were fulfilled (Table 2).

**Table 1:** Timeline of the clinical course.

Time	Clinical findings and assessment	Management and outcome
Initial presentation	Three‐day history of tongue discomfort. A solitary, nonpulsatile hemorrhagic bulla measuring approximately 1.5 × 1.0 cm was present on the right ventral tongue. No other oral or cutaneous lesions, unexplained bruising, hematomas, or airway symptoms were identified.	A clinical diagnosis of ABH was made. The patient was reassured and advised to avoid local trauma and irritating foods.
Four days after presentation	The bulla had ruptured spontaneously, leaving a superficial ulcer measuring approximately 1 × 1 cm.	Conservative management was continued.
Two‐month follow‐up	Complete healing of the ventral‐tongue lesion without clinically visible scarring. No residual palatal ulceration or scarring was present.	No further treatment was required.

Abbreviation: ABH, angina bullosa hemorrhagica.

**Table 2:** Assessment of the present case according to the diagnostic criteria for angina bullosa hemorrhagica proposed by Ordioni et al. [4].

No.	Diagnostic criterion	Assessment in the present case
I	Clinically evident hemorrhagic bulla or erosion with a history of oral mucosal bleeding	Fulfilled: A clinically evident hemorrhagic bulla was documented on the ventral tongue.
II	Exclusively oral or oropharyngeal localization	Fulfilled: No cutaneous or other extraoral mucosal lesions were identified.
III	Palatal localization	Fulfilled: Previous and subsequent palatal episodes were reported.
IV	Triggering event or promoting factor	Not fulfilled: The patient could not recall a precipitating event.
V	Recurrent lesions	Fulfilled: Previous and subsequent oral episodes were reported.
VI	Favorable evolution without scarring within a few days	Fulfilled: Healing without scarring was documented.
VII	Painless lesion, tingling, or burning sensation	Fulfilled: The lesion was painless before rupture, with only tongue discomfort and mild pain after rupture.
VIII	Normal platelet count and coagulation profile	Partially fulfilled: The platelet count was normal, but PT/INR and aPTT results were unavailable.
IX	Negative direct immunofluorescence	Not assessed: Direct immunofluorescence was not performed.

## 3. Discussion

The present case was clinically consistent with ABH, based on the abrupt appearance of a solitary oral hemorrhagic bulla, exclusively oral involvement, previous and subsequent palatal episodes, spontaneous rupture, superficial ulceration, and complete healing without scarring. The ventral tongue is a less typical site than the soft palate and may therefore increase the risk of misdiagnosis [1, 3–5, 15, 19].

Ordioni et al. [4] proposed nine diagnostic criteria for ABH, with six considered sufficient for diagnosis provided that the first two are present. In the present case, the diagnosis was supported by a clinically evident oral hemorrhagic bulla, exclusively oral involvement, recurrent episodes including the classic palatal site, minimal symptoms, and healing without scarring. Although no precipitating event was recalled, this does not exclude minor trauma, because trivial mechanical or thermal insults may be easily overlooked. The nonkeratinized mucosa of the ventral tongue may also be particularly susceptible to unrecognized shearing forces, consistent with the proposed pathogenesis of ABH.

The patient was not taking antiplatelet or anticoagulant medication and had undergone dental extraction without documented bleeding complications. Collectively, these findings lowered but did not eliminate suspicion of an underlying bleeding or coagulation disorder. Baseline coagulation studies should be obtained even when the presentation is solitary and clinically characteristic, and their unavailability in this case is acknowledged as a limitation. Nevertheless, because ABH has no specific confirmatory laboratory test, the characteristic clinical presentation and course supported the diagnosis.

Diabetes mellitus and hypertension, both present in this patient, have been frequently reported among patients with ABH [5, 6, 15–17]. However, the available evidence is observational, and no causal or etiopathogenic relationship has been established [4]. These conditions should therefore be regarded as reported associations rather than confirmed predisposing factors.

Although tongue and floor‐of‐mouth involvement is uncommon, both sites are well documented. In the largest recent multi‐institutional series, Silva‐Cunha et al. [5] reported 23 cases, including six involving the tongue border and one involving the floor‐of‐mouth. Other reported cases of ABH include lateral tongue involvement [9, 19], recurrent tongue involvement [15], floor‐of‐mouth involvement [20], recurrent oral mucosal/tongue involvement [17], food‐triggered pharyngeal lesions [11], and ventral tongue/floor‐of‐mouth presentations [16]. More recently, Khajehzadeh [21] reported ABH of the ventral tongue in a patient with inhaled corticosteroid use and chewing‐related trauma; the lesion resolved within 7 days without scarring following symptomatic management. These reports confirm that tongue and floor‐of‐mouth lesions fall within the clinical spectrum of ABH, even though they are less common than palatal lesions (Table 3) [5, 9, 15, 16, 19–21].

**Table 3:** Selected published angina bullosa hemorrhagica cases involving the tongue or floor‐of‐mouth.

Study	Site	Key clinical feature	Outcome/teaching point
Onda et al. [19]	Right lateral tongue	Recurrent episodes noticed while eating; normal blood tests.	Ruptured in 1–2 days and healed without scarring in about 2 weeks.
Nandini et al. [15]	Dorsal tongue with prior ventral tip/right lateral lesions	Recurrent tongue ABH in a patient with diabetes and hypertension.	Supports tongue recurrence as a recognized ABH pattern.
Silva‐Cunha et al. [5]	Tongue border (six cases); floor‐of‐mouth (one case)	Largest recent series documenting nonpalatal sites.	Confirms tongue/floor‐of‐mouth lesions are established, not exceptional, within the ABH spectrum.
Raizada et al. [20]	Floor‐of‐mouth	Cyclical episodes temporally related to progesterone changes.	Illustrates a rare floor‐of‐mouth presentation with spontaneous regression.
Sachdeva et al. [16]	Ventral tongue; floor‐of‐mouth	Retrospective series including ventral tongue and floor‐of‐mouth cases.	Shows that nonpalatal ABH continues to be documented in recent literature.
Khajehzadeh [21]	Ventral tongue	Painful recurrent lesion associated with chewing hard food in a patient with a history of asthma and inhaled corticosteroid use.	Pain improved with benzydamine and chlorhexidine mouth rinses, and complete healing without scarring occurred within 7 days.
Satici et al. [9]	Lateral tongue	Trauma‐associated lesion after eating sunflower seeds.	Healed without scarring after conservative/limited procedural management.

Abbreviation: ABH, angina bullosa hemorrhagica.

The differential diagnosis of a solitary hemorrhagic bulla or postbullous erosion on the tongue includes traumatic hematoma, thrombocytopenia and other bleeding disorders, vascular malformation, pemphigus vulgaris, mucous membrane pemphigoid, linear IgA disease, epidermolysis bullosa acquisita, bullous lichen planus, and less commonly oral amyloidosis [3, 4, 6, 10, 13, 19]. Traumatic hematomas are usually associated with a more evident history of injury, whereas vascular lesions tend to persist and may be compressible, blanching, or pulsatile. Immunobullous disorders are more likely to produce persistent, multifocal, or recurrent lesions involving additional oral, cutaneous, or other mucosal sites. Hematologic and coagulation disorders may be accompanied by petechiae, unexplained bruising, prolonged bleeding, or abnormal laboratory results. Several features favored ABH over these alternatives: The lesion was confined to the oral cavity, its course was abrupt and self‐limiting, and similar palatal episodes had occurred both before and after it. Nonetheless, a complete blood count with platelet count and a coagulation profile should be obtained in patients with suspected ABH to exclude thrombocytopenia, coagulopathy, or occult hematologic disease. Biopsy and direct immunofluorescence may be considered selectively for lesions that are atypical, multifocal, persistent, or diagnostically doubtful [4, 6, 10, 13]. Given the characteristic clinical presentation and self‐limiting course in the present case, these investigations were not performed.

Histopathologic findings in ABH are usually nonspecific, but reported features include a subepithelial hemorrhagic bulla with extravasated erythrocytes and a mild inflammatory infiltrate. Ruptured lesions show nonspecific ulceration. Direct immunofluorescence is typically negative [1, 3–5].

Management of ABH is usually conservative [8, 14–18]. Patients are advised to avoid local trauma and irritating foods, particularly hard, hot, or sharp foods that may precipitate new lesions [8, 11, 15, 16, 18]. Symptomatic care may include anti‐inflammatory or antiseptic mouth rinses to reduce discomfort and the risk of secondary infection after rupture [8, 9, 11, 15, 16]. In many cases, the intact bulla ruptures spontaneously, followed by healing without scarring [1, 3–5, 8, 15–18]. However, large lesions in the soft palate, uvula, pharynx, or other posterior sites may threaten the airway and require close observation or drainage [7, 8, 11].

The present report documents an uncommon ventral‐tongue presentation through sequential photographs of the intact hemorrhagic bulla, postrupture ulceration, and complete healing without scarring. The principal limitation is the absence of an available coagulation profile during or after the active episode. In addition, the previous and subsequent palatal episodes were reported retrospectively and were not examined during their active stages. Nevertheless, the characteristic clinical appearance, recurrent oral pattern, and self‐limiting course strongly supported the clinical diagnosis of ABH and emphasized the importance of recognizing this condition at less typical oral sites.

## 4. Conclusion

ABH should be considered in the differential diagnosis of solitary hemorrhagic bullae and postbullous erosions of the tongue, including the ventral tongue. Although the soft palate remains the prototypical site, published series confirm that tongue and floor‐of‐mouth presentations are part of the disease spectrum. ABH is primarily diagnosed clinically; however, a complete blood count with platelet count and baseline coagulation studies should be obtained to exclude an underlying hematologic or coagulation disorder. Biopsy with direct immunofluorescence may be considered selectively for persistent, atypical, or extraoral presentations.

**Nomenclature**
ABH: angina bullosa hemorrhagica
IgA: immunoglobulin A

## Author Contributions

**Sara Akeel:** conceptualization, data curation, investigation, methodology, resources, visualization, writing – original draft, writing – review and editing.

## Funding

No funding was received for this manuscript.

## Disclosure

The author has read and approved the final manuscript. Sara Akeel, as corresponding author and guarantor, had full access to all data and takes responsibility for their integrity and the accuracy of the report. All cited references were checked against PubMed and publisher records; none has been retracted.

## Ethics Statement

This report describes a single anonymized clinical case without a research intervention; formal ethics committee approval was therefore not sought.

## Consent

Written informed consent was obtained from the patient for publication of this case report and the accompanying clinical images.

## Conflicts of Interest

The author declares no conflicts of interest.

## Supporting information


**Supporting Information** Additional supporting information can be found online in the Supporting Information section.

## Data Availability

The data that support the findings of this study are available on request from the corresponding author. The data are not publicly available due to privacy or ethical restrictions.
