# Supplementary material for: Angina Bullosa Hemorrhagica of the Ventral Tongue: A Case Report and Literature Review
Source: Case Rep Dent. 2026 Aug 31;2026:4477755. doi: 10.1155/crid/4477755 (PMC13527860; doi:10.1155/crid/4477755)
Supplement: Supplementary file 1 [file CRID-2026-4477755-s001.pdf]

CARE case report guidelines

CARE Checklist of information to include when writing a case report

(cc) BY-NC-ND

Topic	Item	Checklist item description	Reported on Line
Title	1	The diagnosis or intervention of primary focus followed by the words "case report"	1-2
Key Words	2	2 to 5 key words that identify diagnoses or interventions in this case report, including "case report"	32-33
Abstract (no references)	3a	Introduction: What is unique about this case and what does it add to the scientific literature?	17-19, 27-29
	3b	Main symptoms and/or important clinical findings	19-23
	3c	The main diagnoses, therapeutic interventions, and outcomes	23-26
	3d	Conclusion—What is the main "take-away" lesson(s) from this case?	27-31
Introduction	4	One or two paragraphs summarizing why this case is unique (**may include references**)	48-56
Patient Information	5a	De-identified patient specific information.	58, 61-65
	5b	Primary concerns and symptoms of the patient.	58-60
	5c	Medical, family, and psycho-social history including relevant genetic information	61-65
	5d	Relevant past interventions with outcomes	63, 88-89
Clinical Findings	6	Describe significant physical examination (PE) and important clinical findings.	66-73
Timeline	7	Historical and current information from this episode of care organized as a timeline	93 (Table 1)
Diagnostic Assessment	8a	Diagnostic testing (such as PE, laboratory testing, imaging, surveys).	66-73, 86-90
	8b	Diagnostic challenges (such as access to testing, financial, or cultural)	86-90
	8c	Diagnosis (including other diagnoses considered)	74-92, 98-100, 143-158
	8d	Prognosis (such as staging in oncology) where applicable	79-82, 170-177
Therapeutic Intervention	9a	Types of therapeutic intervention (such as pharmacologic, surgical, preventive, self-care)	77-79
	9b	Administration of therapeutic intervention (such as dosage, strength, duration)	Not applicable
	9c	Changes in therapeutic intervention (with rationale)	Not applicable
Follow-up and Outcomes	10a	Clinician and patient-assessed outcomes (if available)	79-86, 93
	10b	Important follow-up diagnostic and other test results	81-90
	10c	Intervention adherence and tolerability (How was this assessed?)	Not applicable
	10d	Adverse and unanticipated events	80-85
Discussion	11a	A scientific discussion of the strengths AND limitations associated with this case report	170-177
	11b	Discussion of the relevant medical literature **with references**.	101-169
	11c	The scientific rationale for any conclusions (including assessment of possible causes)	102-125, 143-177
	11d	The primary "take-away" lessons of this case report (without references) in a one paragraph conclusion	178-186
Patient Perspective	12	The patient should share their perspective in one to two paragraphs on the treatment(s) they received	58-60 (limited)
Informed Consent	13	Did the patient give informed consent? Please provide if requested 199-200	Yes ☒ No ☐
